# Supplementary material for: Body composition as a potential biomarker of recurrence risk in patients with triple-negative breast cancer
Source: Breast Cancer Res Treat. 2025 Mar 11;211(3):627–35. doi: 10.1007/s10549-025-07675-w (PMC12031647; doi:10.1007/s10549-025-07675-w)
Supplement: Supplementary file 1 — Supplementary file1 (DOCX 403 KB) [file 10549_2025_7675_MOESM1_ESM.docx]

**Body composition as a novel biomarker of recurrence risk in patients with triple-negative breast cancer**

De Vis JB^1^, Wang C ^2^, Nguyen KV^3^, Sun L^4^, Jia B^3^, Sherry AD^5^, Alford-Holloway MN^6^, Balbach ML^6^, Koyama T^4^, Chakravarthy AB^1^, Rafat M^1,7,8*^

^1^Department of Radiation Oncology, Vanderbilt-Ingram Cancer Center, Nashville, TN

^2^Division of Epidemiology, Department of Medicine, Vanderbilt Epidemiology Center, Nashville, TN

^3^Vanderbilt University School of Medicine, Nashville, TN

^4^Department of Biostatistics, Vanderbilt University Medical Center, Nashville, TN

^5^Department of Radiation Oncology, MD Anderson Cancer Center, Houston, TX

^6^Department of Medicine, Vanderbilt University Medical Center, Nashville, TN

^7^Department of Chemical and Biomolecular Engineering, Vanderbilt University, Nashville, TN

^8^Department of Biomedical Engineering, Vanderbilt University, Nashville, TN

*Author for correspondence: Marjan Rafat, Engineering and Science Building, Rm. 426, Vanderbilt University, Nashville, TN 37212. Phone: (615) 343-3389, Fax: (615) 343-7951, E-mail: marjan.rafat@vanderbilt.edu

**Supplemental Figures**


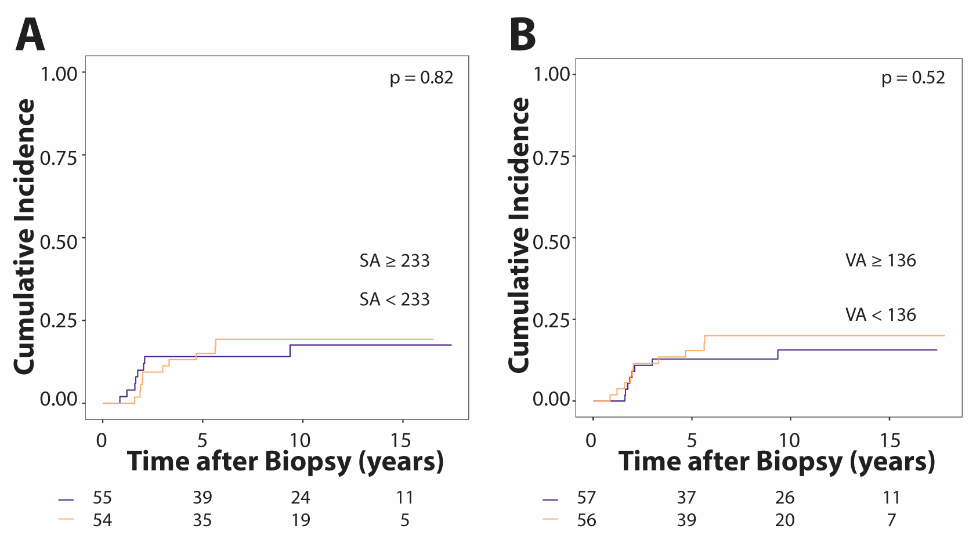


**Supplemental Fig. S1** Cumulative incidence of distant recurrence stratified using median anthropometric and body composition measures. Distant recurrence over time is shown for (**A**) subcutaneous adiposity (SA; blue line, SA < 233 cm^3^; orange line, SA ≥ 233 cm^3^), and (**B**) visceral adiposity (VA; blue line, VA < 136 cm^3^; orange line, VA ≥ 136 cm^3^). At risk subjects are indicated along the x-axis.

**
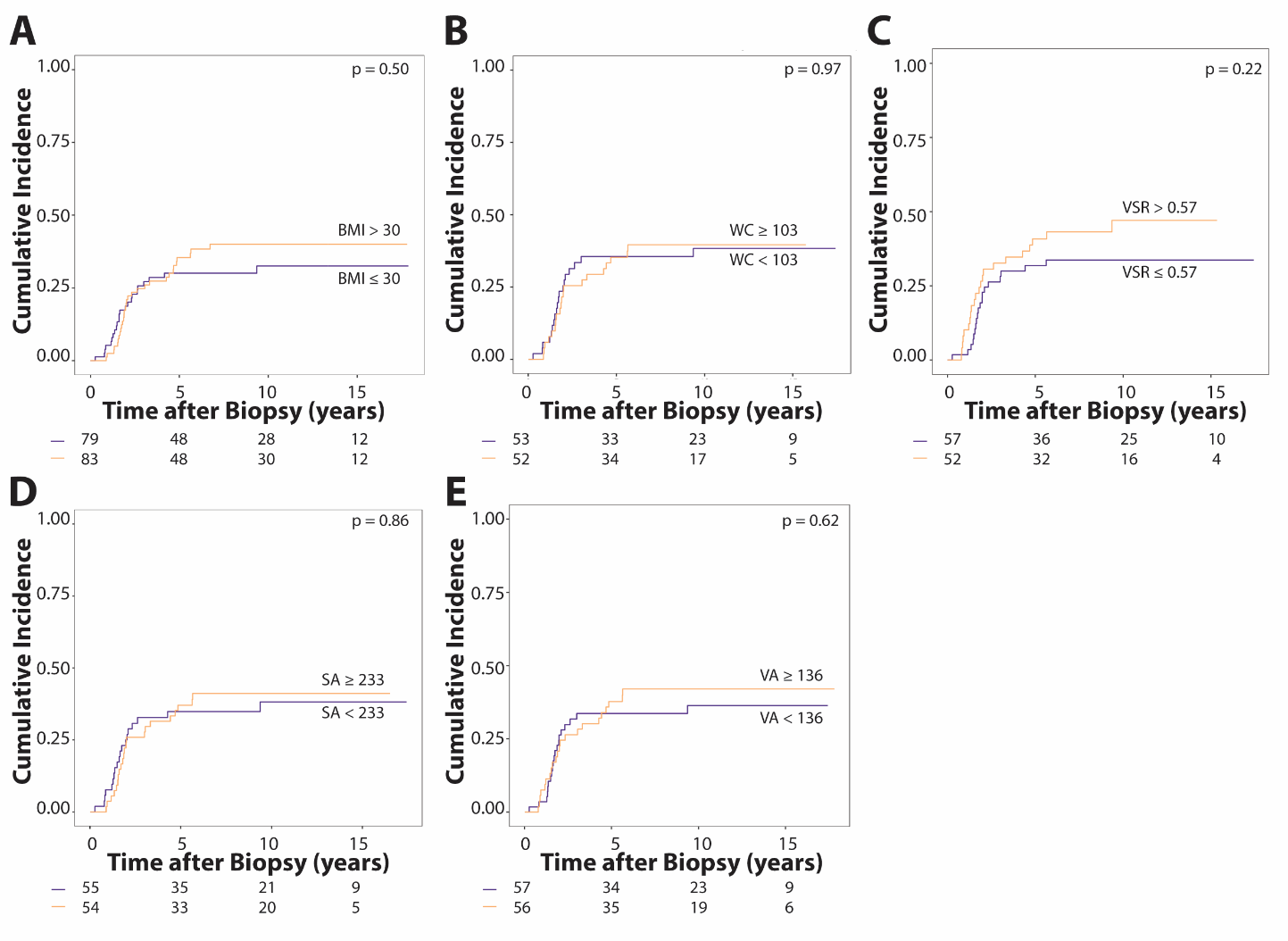
**

**Supplemental Fig. 2** Cumulative incidence of locoregional recurrence stratified using median anthropometric and body composition measures. Locoregional recurrence over time is shown for (**A**) body mass index (BMI; blue line, BMI ≤ 30 kg/m^2^; orange line, BMI > 30 kg/m^2^), (**B**) waist circumference (WC; blue line, WC < 103 cm; orange line, WC ≥ 103 cm), (**C**) visceral-to-subcutaneous adiposity ratio (VSR; blue line, VSR ≤ 0.57; orange line, VSR > 0.57), (**D**) subcutaneous adiposity (SA; blue line, SA < 233 cm^3^; orange line, SA ≥ 233 cm^3^), and (**E**) visceral adiposity (VA; blue line, VA < 136 cm^3^; orange line, VA ≥ 136 cm^3^). At risk subjects are indicated along the x-axis.
